# Infectious Fevers and Diseases in London

**Published:** 1887-02-19

**Authors:** 


### INFECTIOUS FEVERS AND DISEASES IN LONDON.

BY "PUBLIC HEALTH."

IT will be seen from the accompanying Table that the prevalent disease in London at the present time is scarlet fever, of which 162 cases were admitted into the hospitals of the Metropolitan Asylums Board during the four weeks ending January 28th. Numerous as these cases are, there is, nevertheless, an indication that the disease is subsiding, and it may be anticipated that this decline will be maintained until the autumn of the present year. London still remains unusually free from small-pox, but four cases having been admitted into these hospitals during the month. When small-pox is likely to assume epidemic proportions it usually indicates its tendency to spread in the early part of the year. So far as the present year is concerned, we may hope that no undue prevalence will tax the resources of our local authorities. Enteric fever, too, shows but little activity, only sixteen cases being admitted during January. The one disease which creates most reason for anxiety is typhus fever, which, after prolonged absence from the Metropolis, is showing a tendency to return,—one case was admitted from Camberwell, three from Chelsea, and three from St. Saviour's, Southwark. Doubtless the destitution of the present winter has the effect of rendering the poor especially susceptible to this malady; and though London has, by a more active sanitary administration, for a time freed itself from this disease, it has been constantly exposed to its importation from other towns, which have served to nurture it, and which are probably serving as centres for its dissemination at the present time. The prevention of typhus must be undertaken in these places, and should engage the attention of the Central Authority.

The unusual record of the removal of a case of relapsing fever necessarily excites comment. It is now nearly twenty years since this disease visited London as an epidemic, and the occurrence of single cases is a rare event. We would gladly receive information from the hospital authorities as to whether the sufferer exhibited the usual symptoms which are diagnostic of this disease.

*Return of Cases admitted into the Hospitals of the Metropolitan Asylums Board during the four weeks ending January 28th, 1887.*

| SANITARY DISTRICT. | Small-Pox. | Scarlet Fever. | Enteric Fever. | Typhus Fever. | Relapsing Fever. |
|---|---|---|---|---|---|
| Bethnal Green | .. | 7 | 1 | .. | .. |
| Camberwell | .. | 9 | .. | 1 | .. |
| Chelsea | .. | 3 | 1 | 3 | .. |
| Clerkenwell | .. | 11 | .. | .. | .. |
| Fulham | .. | 4 | .. | .. | .. |
| Greenwich | .. | 3 | 1 | .. | .. |
| Hackney | .. | 11 | 1 | .. | .. |
| Hammersmith | .. | .. | 1 | .. | .. |
| Hampstead | .. | 4 | .. | .. | .. |
| Holborn | .. | 5 | .. | .. | .. |
| Islington | .. | 8 | 1 | .. | .. |
| Kensington | .. | 1 | 1 | .. | .. |
| Lambeth | .. | 9 | .. | .. | .. |
| Limehouse | .. | 1 | .. | .. | .. |
| Newington | .. | 6 | .. | .. | .. |
| Paddington | .. | 9 | .. | .. | .. |
| Poplar | .. | 2 | .. | .. | .. |
| Rotherhithe | .. | .. | 1 | .. | .. |
| Shoreditch | .. | 4 | 1 | .. | .. |
| St. George's, Hanover Sq. | .. | 5 | .. | .. | .. |
| " in the East | 2 | 3 | 2 | .. | .. |
| " Bloomsbury | .. | .. | 1 | .. | .. |
| St. James's, Westminster | .. | 1 | .. | .. | .. |
| St. Luke's | .. | 11 | 1 | .. | .. |
| St. Martin's-in-the-Fields | .. | 4 | .. | .. | .. |
| St. Marylebone | .. | 3 | 1 | .. | .. |
| St. Pancras | .. | 14 | .. | .. | .. |
| St. Saviour's | .. | 3 | 2 | 3 | .. |
| Wandsworth | .. | 8 | .. | .. | .. |
| Westminster | .. | 10 | .. | .. | .. |
| Whitechapel | 1 | 3 | .. | .. | 1 |
| Port Sanitary Authority | 1 | .. | .. | .. | .. |
| TOTALS | 4 | 162 | 16 | 7 | 1 |

